# Cloning and characterization of *TaVIP2* gene from *Triticum aestivum* and functional analysis in *Nicotiana tabacum*

**DOI:** 10.1038/srep37602

**Published:** 2016-11-18

**Authors:** Pei Zhao, Ke Wang, Zhishan Lin, Wei Zhang, Lipu Du, Yunlong Zhang, Xingguo Ye

**Affiliations:** 1 Institute of Crop Science, Chinese Academy of Agricultural Sciences, Beijing 100081, P.R. China

## Abstract

Wheat is recalcitrant to genetic transformation. A potential solution is to manipulate the expression of some host proteins involved in T-DNA integration process. VirE2 interacting protein 2 (VIP2) plays an important role in T-DNA transport and integration. In this study, a *TaVIP2* gene was cloned from common wheat. Southern blot and allele-specific polymerase chain reaction (AS-PCR) combined with an online chromosomal location software tool revealed that three *TaVIP2* genes were located on wheat chromosomes 1AL, 1BL, and 1DL. These three homoeoallelic *TaVIP2* genes all contained 13 exons and 12 introns, and their coding sequences were the same; there were a few single nucleotide polymorphisms (SNPs) among the three genes. The heterologous expression of the *TaVIP2* gene in tobacco led to enhancement of the *Agrobacterium*-mediated transformation efficiency up to 2.5-fold. Transgenic tobacco plants expressing *TaVIP2* showed enhanced resistance to powdery mildew. Further quantitative real-time PCR (qRT-PCR) revealed that overexpression of *TaVIP2* in transgenic tobacco up-regulated the expression of an endogenous gene, *NtPR-1*, which likely contributed to powdery mildew resistance in transgenic tobacco. Our study indicates that the *TaVIP2* gene may be highly useful in efforts to improve *Agrobacterium*-mediated transformation efficiency and to enhance powdery mildew resistance in wheat.

Plant VirE2 interacting proteins (VIPs), a group that includes VIP1 and VIP2, are host proteins that specifically interact with the VirE2 protein of *Agrobacterium*. Once attached to plant cells, *Agrobacterium* immediately exports T-DNA and some effector proteins such as VirE2, VirD2, and VirF, into the host via a type IV secretion system. Among these proteins, VirD2 and VirE2 may combine with T-DNA to protect the foreign DNA from being degraded in plant cells^1^. Although the plant initiates its defense system(s) against invasion^1^, there are still some plant proteins, such as VIP1, VIP2, KAPa, PP2C, and VBF^2^, that actually assist the foreign proteins with the cytoplasm transportation and nuclear import of the T-DNA complex. VIP1 and VIP2 were first identified from an *Arabidopsis* cDNA library using VirE2 as a bait protein in yeast two-hybrid assays^3^.

VIP1 is composed of 261 amino acids and contains a leucine zipper domain (a plant basic-zipper protein (bZIP)) that is homologous to most plant bZIP proteins containing a nuclear localization signal (NLS) in the conserved region of the leucine zipper domain^4^. After antisense VIP1 cDNA of *Arabidopsis* was transferred into tobacco with an *Agrobacterium*-mediated technique, the transgenic plants displayed strong resistance to *Agrobacterium*-mediated retransformation. The expression level of VIP1 decreased, and VirE2 did not enter the nucleus in the transgenic tobacco plants, blocking the early process of T-DNA transportation into host cells upon *Agrobacterium* infection^5^. In contrast, overexpression of *AtVIP1* in transgenic tobacco plants dramatically enhanced T-DNA transformation efficiency when retransformed with *Agrobacterium*^6^. These results demonstrated that VIP1 plays a vital role during the nuclear import of T-DNA in the *Agrobacterium*-mediated transformation process. When the VIP2-silenced transgenic tobacco and *Arabidopsis* plants were retransformed by *Agrobacterium* or biolistic particles, the stable transformation efficiencies declined, but their transient transformation efficiencies by the pathogen and stable transformation efficiencies by bombardment were not affected^7^. This finding suggested that VIP2 is also involved in the integration of T-DNA into the plant genome. In a VIP2 deficient *Arabidopsis* mutant, the accumulation of histone mRNA decreased when the mutant was transformed by *Agrobacterium*, indicating that the mutant’s resistance to *Agrobacterium* transformation might be due to the deficiency of VIP2. The lack of VIP2 affected histone binding to T-DNA and suppressed the integration of T-DNA to some extent. Sequence analysis revealed that VIP2 had a conserved NOT (negative on the TATA-less) domain at its carboxyl terminus. Proteins with a NOT domain, such as NOT2/NOT3/NOT5, were initially identified in yeast. These are known to enhance the transcriptional function of promoters lacking of a TATA element^8^. Therefore, NOT proteins possess dual functions and can positively or negatively modulate the expression level of target genes^9^. However, the functions of most proteins with NOT domains in plants remain unclear. Based on the NOT protein homologous relationship between plants and yeast or animals, plant VIP2 can be inferred to have a function in regulating gene expression. Affymetrix microarray analysis comparing a *vip2* mutant and wild type *Arabidopsis* revealed many differentially expressed candidate genes following *Agrobacterium* infection^7^.

It is clear that plant VIP1 and VIP2 both play roles in T-DNA nuclear import and chromosome integration and function to modulate host gene expression. Wheat is one of the most important crops in the world, grown in a global area of about 220 million hectares. Its yield and quality are closely associated with the sustainable development of communities and economies^10^. Wheat production is seriously damaged by various biotic and abiotic stresses such as powdery mildew, rusts, scab, drought, and salt. Although genetic engineering has been developed to tackle such problems^11^, wheat has lagged behind other major grain crops largely due to the lack of efficient transformation techniques^12^. Therefore, it is necessary to isolate and characterize the orthologous genes of *Arabidopsis VIP1* and/or *VIP2* in wheat for potential use in the enhancement of *Agrobacterium*-mediated transformation efficiency and disease resistance. A wheat gene, *TaVIP2*, was isolated and characterized in this study. Its copy number and chromosome location in the wheat genome were also determined. The primary biological function of *TaVIP2* was further investigated in tobacco. Our work expands the knowledge and genetic resources and information available for wheat improvement.

## Results

### *TaVIP2* cloning and chromosomal location analysis

Using the *AtVIP2* gene (NM125363) in *Arabidopsis* as a seed sequence to blast NCBI databases, one EST AK331382 was found in wheat. After analyzing this sequence, a putative full sequence of *TaVIP2* was acquired. Primers were designed according to the assembled sequence to amplify the complementary DNA (cDNA) sample from wheat line CB037. A 1900 bp fragment was amplified (see Supplementary Fig. S1). Sequencing analysis revealed that the ORF of *TaVIP2* was 1839 bp in length. At the protein level, *TaVIP2* was 612 amino acids in length and had only 48% similarity with AtVIP2. We registered *TaVIP2* with NCBI with the accession number KF752430.

**Figure 1:**
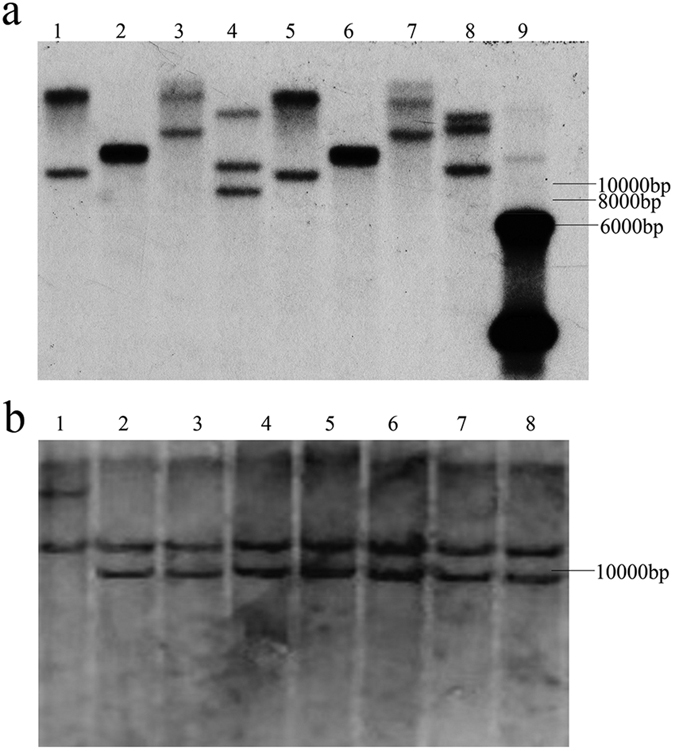
Copy number and chromosomal location analysis of TaVIP2 in wheat genome by Southern blot. Three copies of *TaVIP2* genes were present in the genome of common wheat revealed by various enzymes (**a**); genomic DNA of wheat line Luivo was digested by *Eco*RV, *Sac*I, *Kpn*I, and *Dra*I (lanes 1–4); genomic DNA of wheat line CB037 was digested by *Eco*RV, *Sac*I, *Kpn*I, and *Dra*I (lanes 5–8); plasmid DNA containing *TaVIP2* was used as check (lane 9); there is no restriction site for *Eco*RV, *Sac*I, *Kpn*I, or *Dra*I in the genomic DNA sequence of *TaVIP2*; the full length cDNA of *TaVIP2* labeled with Digoxigenin was used as the probe. One *TaVIP2* gene was proved to be located on chromosome 1 A using seven durum wheat substitution lines analyzed by Southern blot (**b**); durum wheat substitution lines 1D(1 A), 2D(2 A), 3D(3 A), 4D(4 A), 5D(5 A), 6D(6 A), and 7D(7 A) in which a pair of A-genome chromosomes in durum wheat were replaced by its corresponding pair of D-genome chromosome from hexaploid wheat cultivar CS (lanes 1–7); receptor durum wheat line of Langdon was used as control (lane 8); plant DNA samples were digested with *Dra*I and then hybridized with the *TaVIP2* coding sequence labeled with Digoxigenin as the probe; Substitution line 1D(1 A) showed a different band from the parent durum wheat line Langdon and the other six durum wheat substitution lines.

Southern blot analysis of the two wheat materials CB037 and Luivo revealed that three genes of a family with *TaVIP2* were present in the hexaploid wheat genome; these were located on the A, B, and D genomes (Fig. 1a). Further, genomic DNA of seven durum wheat and D genome chromosome substitution lines of 1D(1 A) to 7D(7 A) were digested with *Dra*I for Southern blot analysis. Six lines showed the same band patterns as the parent durum wheat line Langdon, whereas the 1D(1 A) line showed a different band pattern (Fig. 1b). This indicated that one copy of *TaVIP2* was located on chromosome1A. According to the orthologous relationship of wheat genes among the different genomes, the other two copies of *TaVIP2* were predicted to be located on chromosomes 1B and 1D.

To verify the chromosome location of the three homoeoallelic *TaVIP2* genes by Southern blot and orthologous gene speculation, three pairs of primers for AS-PCR that only can amplify the parallel regions of the three *TaVIP2* genes were designed for each of the three homoeoallelic *TaVIP2* genes from the A, B(S), and D, genomes (see Supplementary Table S1). When the two sets of durum wheat substitution lines of 1D(1 A) to 7D(7 A) and 1D(1B) to 7D(7B) were used as templates for the amplification with the specific primers to the allele on the A genome, the substitution lines of 2D(2 A) to 7D(7 A) and 1D(1B) to 7D(7B), as well as Langdon, showed the 802 bp specific band but substitution line 1D(1 A) did not show the band (see Supplementary Fig. S2a). When amplified by the specific primers to the allele on B genome, 1D(1 A) to 7D(7 A) and 2D(2B) to 7D(7B), a 499 bp specific band was present as the parent Langdon; this band was not present in substitution line 1D(1B) (see Supplementary Fig. S2b). When amplified by the specific primers to the allele on D genome, only substitution lines 1D(1 A) and 1D(1B) showed the 447 bp specific band; the other lines, as well as Langdon, did not display this band (see Supplementary Fig. S2c). By using an online software tool for chromosomal location analysis of plant genes (https://urgi.versailles.inra.fr/blast/blast.php), the three *TaVIP2* genes were further confirmed to be located on chromosomes 1AL, 1BL, and 1DL.

### Structure and phylogenic analysis of *TaVIP2*

**Figure 2:**
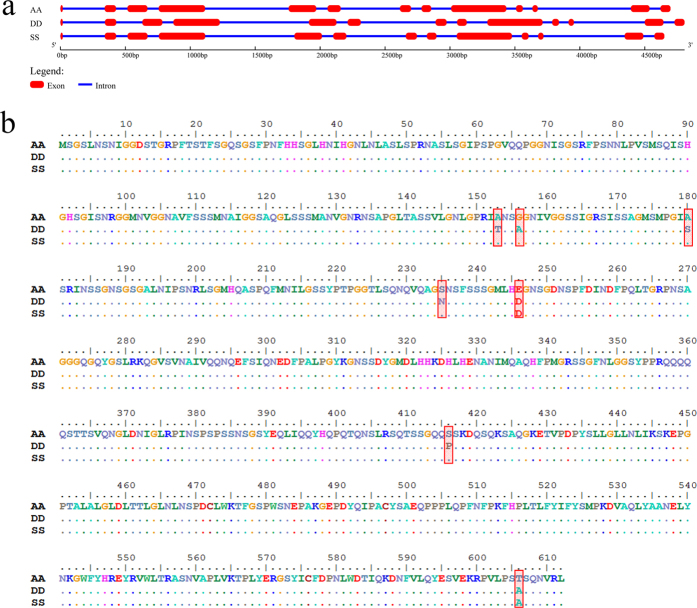
Comparison of the gene structures and predicted protein sequences of the three *TaVIP2* genes in wheat. Three homoeoallelic *TaVIP2* genes from the A, D, and S genomes (donor of wheat B genome) were 4698 bp, 4806 bp, and 4651 bp, in length, respectively (**a**); The differences in the amino acid sequences among the TaVIP proteins encoded by the three homoeoallelic *TaVIP2* genes of the A, B(S), and D, genomes are marked in red frames (**b**).

To obtain the sequences and clarify the molecular structures of the three *TaVIP2* genes in wheat, the gDNA and cDNA prepared from three wild relatives of common wheat (with genomes A, S, and D) were used as a template for the amplification of *TaVIP2*. Among the three ancestral genomes of wheat, S the genome from *Aegilops speltoides* is the donor of the wheat B genome, and is therefore denoted as B(S) in the following text. After sequencing of the PCR products, three sequences (gDNA and their corresponding cDNA) were obtained for the A, B(S), and D, genomes, respectively. Comparison of the gDNA and corresponding cDNA sequences of each *TaVIP* gene showed that all three *TaVIP2* genes contained 13 exons and 12 introns (Fig. 2a). The 13 exons of *TaVIP2* were the same in size among the three wheat genomes (see Supplementary Table S2). However, there were 31 single nucleotide polymorphisms (SNPs) among the cDNA sequences of the three genes of the *TaVIP2* family from the A, B(S), and D genomes, 12 SNPs between the A and B(S) genomes, 26 SNPs between the A and D genomes, and 25 SNPs between the B(S) and D genomes (see Supplementary Fig. S3). The cDNA sequences of *TaVIP2* from the A and B(S) genomes were more similar to each other than were any other comparative pairing. The predicted *TaVIP2* protein sequences encoded by the three *TaVIP2* genes were almost identical, with the exception of seven amino acid differences at positions 153, 156, 180, 235, 246, 416, and 606 (Fig. 2b). However, regarding the 12 introns among the three genes of *TaVIP2* there were significant differences for both intron length and intron sequence among the *TaVIP2* genes (Fig. 2a and see Supplementary Table S2). For example, the length of the eleventh intron from the A genome was 718 bp, 547 bp from the B(S) genome, and 626 bp from the D genome (see Supplementary Table S2). Genetic variation of the three *TaVIP2* genes in common wheat was caused by the intron; there were nearly no changes in their exonic sequences.

To investigate the phylogenetic relationships of VIP2 genes among different plant species, the complete or partial coding sequences of 28 putative plant *VIP2* genes obtained from GenBank by BLAST analysis, as well as the *TaVIP2* cloned in the present study were used to construct a phylogenetic tree. The phylogenetic tree was apparently clustered into two branches (see Supplementary Fig. S4), with one branch containing the genes from monocotyledonous plants, and another branch covering eudicot genes. Six candidate *VIP2* genes of the *Gramineae* were clustered into one branch; this branch, included *VIP2* genes from *Oryza sativa*, *Setaria italic*, *Sorghum bicolor*, *Brachypodium distachyon*, *Triticum uratu*, and *Triticum aestivum*. Further, the VIP2 genes from *Triticum uratu* and *Triticum aestivum* were clustered into a subgroup, suggesting that they may have similar structural features and a close phylogenetic relationship.

### Functional characterization of *TaVIP2* in transgenic tobacco

**Figure 3:**
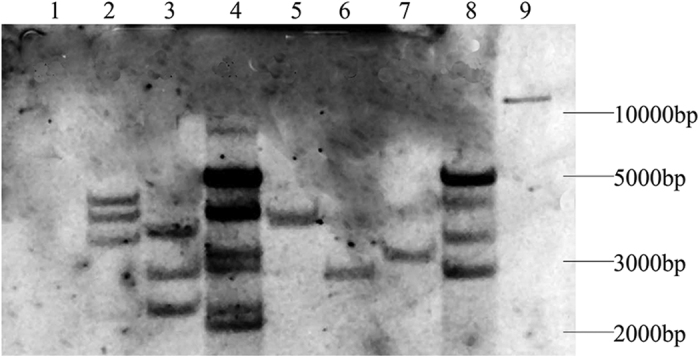
Southern blot analysis of transgenic tobacco plants overexpressing *TaVIP2*. Lane 1 is for a negative transgenic tobacco plant, lanes 2–8 are for *TaVIP2* transgenic tobacco lines TV2-14, TV2-21, TV2-41, TV2-49, TV2-58, TV2-12, and TV2-13, respectively, and lane 9 is for a plasmid containing the *TaVIP2* gene; the full length coding sequence of *TaVIP2* labeled with Digoxigenin was used as the probe for Southern blot analysis, in which tobacco genomic DNA was digested with *Hin*dIII.

Because the ORF sequences of the three *TaVIP2* genes in wheat were very similar to each other, the *TaVIP2* gene was amplified only from the cDNA of the wheat A genome and subcloned into the a modified pBI121 expression vector (see Supplementary Fig. S5). Next, the *TaVIP2* gene was transferred into tobacco by *Agrobacterium*-mediated transformation. In total, 50 transgenic plants were confirmed by PCR with specific primers for the *TaVIP2* gene (see Supplementary Fig. S6). Southern blot using the Digoxigenin-labeled full length cDNA sequence of *TaVIP2* as a probe confirmed that the *TaVIP2* gene was integrated into the tobacco genome; the number of integrated points ranged from one to six (Fig. 3).

The transgenic tobacco plants with a single integration of the *TaVIP2* gene (confirmed by Southern blot) as well as the negative transgenic tobacco plants were propagated by tissue culture and then retransformed by *Agrobacterium* harboring a vector with the *GUS* and *bar* genes (see Supplementary Fig. S7). Our results showed that the *TaVIP2* overexpressing tobacco plants TV2-11 and TV2-49 generated more shoots than did negative transgenic tobacco plants under bialaphos selection after *Agrobacterium* infection and co-cultivation (see Supplementary Fig. S8). Screening retransformed transgenic plants through PCR for presence of the *bar* gene revealed that the retransformation efficiencies of TV2-11 and TV2-49 were elevated by 2.1 and 2.5-fold, respectively, compared with negative transgenic tobacco plants (Table 1, see Supplementary Fig. S9). These results indicated that the expression of *TaVIP2* could improve the *Agrobacterium*-mediated transformation efficiency of tobacco to some extent.

### Evaluation of powdery mildew resistance of the transgenic tobacco plants

**Figure 4:**
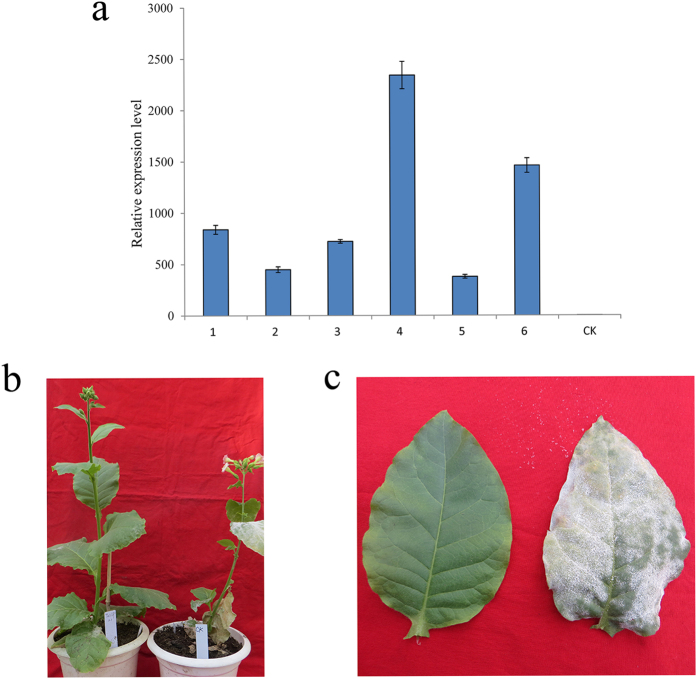
Expression level analysis of *TaVIP2* gene in *TaVIP2* transgenic tobacco plants and their resistance test to powdery mildew. The expression level of *TaVIP2* was revealed by real time PCR, in which samples 1, 2, 3, 4, 5, and 6 represent *TaVIP2* transgenic tobacco lines TV2-11, TV2-12, TV2-13, TV2-14, TV2-21, and TV2-49, respectively, and CK stand for the wild type tobacco NC89. Transgenic tobacco plants exhibited powdery mildew resistance phenotypes (the left one in **b** and **c**), while the wild type tobacco plant (NC89) showed powdery mildew sensitivity phenotypes (the right one in **b** and **c**).

**Figure 5:**
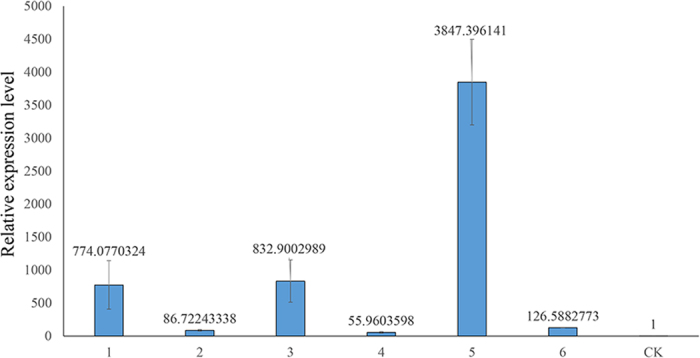
Expression level of pathogenesis–related gene *NtPR-1* in the transgenic tobacco plants expressing *TaVIP2*. Samples 1, 2, 3, 4, 5, and 6 represent *TaVIP2* transgenic tobacco lines TV2-11, TV2-12, TV2-13, TV2-14, TV2-21, and TV2-49, respectively, and CK stands for the wild type tobacco NC89 plant. The expression level of *NtPR-1* was dramatically elevated in the transgenic tobacco lines containing *TaVIP2* compared with the wild type tobacco, especially in the transgenic line TV2-21.

The *TaVIP2* transgenic tobacco plants were subjected to powdery mildew resistance assays; leaves were inoculated with the causal pathogen of powdery mildew in the greenhouse. The expression level of *TaVIP2* was measured with real-time PCR. Under the pathogen stress, all of the transgenic plants showed much higher *TaVIP2* expression than did the control plants (Fig. 4a). The TV2-14 and TV2-49 transgenic plants exhibited extremely high *TaVIP2* expression. The transgenic plants showed resistance to mixed powdery mildew infection with a disease incidence of 0–10% or a resistance grade of 0–3 (no or few powdery mildew colonies were observed on the leaves), while the control plants were severely infected by the pathogen (disease incidence over 80% or resistance grade up to 9) (Fig. 4b and c). Among the transgenic plants, TV2-11, TV2-13, TV2-14, and TV2-49, which contained 1 to 4 copies of *TaVIP2* in their genome (Fig. 3), showed the highest degree of powdery mildew resistance, with resistance grade levels of 0 or 1. To explore the possible mechanism of the enhancement of tobacco resistance to powdery mildew by expressing *TaVIP2*, the expression level of some pathogen resistance related genes, including *NtPR-1*, *NtMla*, *NtHsp*, *NtRAR*, and *NtMAPK*, was measured in the transgenic tobacco plants containing *TaVIP2* using qRT-PCR. Only the *NtPR-1* gene was up-regulated in the *TaVIP2* transgenic tobacco plants as compared with the negative control (Fig. 5). The expression levels of other genes tested were not significantly different in the transgenic plants and the negative transgenic plants (see Supplementary Fig. S10).

## Discussion

Although the first transgenic wheat plant was achieved by biolistic particle bombardment in 1991, *Agrobacterium*-mediated wheat transformation was not successful until 1997, with an efficiency of 1.12-1.15%^13^,^14^. More reports on transgenic wheat using *Agrobacterium*-mediated technology have been published since then. For example, Roundup Ready wheat with herbicide resistance was developed with this technique^15^. Improved transformation efficiency was achieved by using a super expression vector and adding polyamine compounds to co-cultivation media^16^,^17^. Desiccation treatment of infected wheat tissues during co-culturing was found to enhance the transformation efficiency of T-DNA^18^. In addition, *Agrobacterium* mediated and floral dipping transformations were also reported in wheat a few years ago^19^,^20^. However, *Agrobacterium*-mediated transformation of wheat is known to be affected dramatically by the genotypes and physiological status of immature embryo explants. There has been difficulty with repeating transformation results in different laboratories. This situation limits the development of transgenic wheat materials^12^. The need to improve the *Agrobacterium*-mediated transformation efficiency of wheat remains urgent. Manipulating the expression of some host proteins involved in the transportation of T-DNA inside wheat cells to enhance stable transformation efficiency might be a workable strategy to improve transformation efficiency.

*Agrobacterium*, as the most efficient transformation vehicle in plant transformation, has been widely used in genetic engineering studies for many eudicot and some monocotyledonous plants^21^. Transferring target genes into a plant genome by *Agrobacterium* is a complex biological process in which a lot of *Agrobacterium* proteins and plant proteins interact with each other to import, transport, and integrate T-DNA^2^,^22^,^23^. In *Arabidopsis*, some associated host proteins involved in the delivery of T-DNA were identified using various mutants and yeast two hybrids^24^. Those associated host proteins play important roles in nearly every step of the transformation of exogenous genes^2^,^25^. For instance, AGPs, rhicadhesin binding proteins, and vitronectin-like proteins are involved in the attachment of *Agrobacterium* to the surface of plant cells^24^,^26^. The GTPase and BTI proteins help T-DNA and Vir proteins enter the plant cell^27^. Actin, GIP, VIP1, and VIP2 assist T-DNA transportation in the plant protoplasm^23^,^24^. VIP1, VIP2, KAPa, PP2C, and Roc participate in the targeting and importing of T-DNA complexes into the plant nucleus^2^,^5^,^28^,^29^,^30^. Histones, VIP1, Ku80, and VIP2 all perform functions related to the integration of T-DNA into the plant genome^2^,^31^,^32^. Reports have indicated that the expression of VIP1, AGP, VIP2, and H2A from *Arabidopsis* are closely associated with the *Agrobacterium*-mediated transformation efficiency of some model plants such as tobacco and rice^6^,^7^,^33^,^34^,^35^. However, the functions of these proteins in plants recalcitrant to *Agrobacterium* infection, such as wheat and maize, remain as yet undetermined. Thus, some novel proteins or genes associated with T-DNA transformation need to be identified and characterized in plant species recalcitrant to *Agrobacterium* infection.

In our previous study, higher transient expression of the *GUS* gene (up to 81.9% for some genotypes) did not lead to higher stable transformation efficiency in *Agrobacterium* mediated wheat transformation^36^,^37^. This may be due to the low efficiency of T-DNA integration into the plant genome despite the easy importation of T-DNA into host cells observed for these plants. Alien T-DNA might encounter issues in wheat cells such as degradation, inefficient transportation, or low levels of nuclear input, owing possibly to the lack of protection and/or assistance of host proteins. Therefore, further investigating the effectiveness of wheat-related proteins on the integration of T-DNA is necessary to improve stable transformation efficiency mediated by *Agrobacterium*. In this study, the *TaVIP2* from wheat was isolated and expressed in tobacco. Our results demonstrated that overexpression of *TaVIP2* in tobacco lead to increased efficiency in *Agrobacterium*-mediated transformation (see Supplementary Fig. S8, Table 1). This finding has prompted us to over-express *TaVIP2* in wheat. We look forward to evaluating any improvement that such a strategy may cause and are particularly interested in assessing this approach in the widely commercialized wheat varieties that are highly recalcitrant to genetic modification.

*Arabidopsis* VIP1, as a functional transcriptional factor of the basic leucine zipper (bZIP) domain family, activates the expression of many defense-related genes by binding with VIP1 response elements after being phosphoresced by MAPK, and endows plants with resistance to various diseases^30^,^38^. Moreover, it was found that VIP1 also participates in the signal transduction of plant immunity as induced by *Agrobacterium*^39^. However, the function of plant VIP2 on disease resistance has not been reported by other researchers to date. In this study, transgenic tobacco plants expressing wheat *TaVIP2* exhibited almost complete resistance to powdery mildew (Fig. 4b and c). Further investigation showed that the expression level of the *NtPR-1* gene was up-regulated in the transgenic tobacco plants expressing *TaVIP2* (Fig. 5). This result indicated that the expression of *TaVIP2* may have some relationship with the expression of *NtPR-1*. Overexpression of *TaVIP2* may modulate the expression of *NtPR-1*, a pathogenesis resistance-related endogenous gene in tobacco, and endowed transgenic tobacco plants resistance to powdery mildew. A previous study demonstrated that *NtPR-1* in tobacco is an important marker gene of systemic acquired resistance to plant diseases^40^. Expression of the *PR-1* gene from *Wasabia japonica* in tobacco conferred transgenic plants with resistance to *Botrytis cinerea*^41^. The wheat genes *PR-1*, *PR-2*, and *PR-5*, which are homologous to *NtPR-1* have been confirmed to be associated with defensive responses to the powdery mildew pathogen. Our research has discovered that the wheat *TaVIP2* gene bestowed transgenic tobacco plants with resistance to powdery mildew in a manner possibly associated with the up-regulation of the expression of *NtPR-1*, although the underlying mechanism of VIP2 mediated disease resistance in plants needs to be investigated further. Further studies will be needed to evaluate whether or not the overexpression of *TaVIP2* in wheat can improve its resistance to diseases including powdery mildew.

## Materials and Methods

### Plant materials

The tissue-culture-favorable common wheat line CB037 (*Triticum aestivum L*., AABBDD, 2n = 42) developed by our research group was used for the cloning of *TaVIP2*. *Triticum aestivum* variety Luivo, was kindly provided by Dr. Tom Clemente at the University of Nebraska-Lincoln, USA. A set of durum wheat *(Triticum turgidum*, AABB, 2n = 28) and *Aegilops tauschii* (DD, 2n = 14) substitution lines of 1D(1 A) to 7D(7 A) and 1D(1B) to 7D(7B), in which a pair of A-genome or B-genome chromosomes were replaced by corresponding pairs of D-genome chromosomes transferred from hexaploid wheat cv. Chinese Spring (CS)^42^, were used to ascertain the chromosome location of the *TaVIP2*. The substitution lines and their parent durum wheat line Langdon were kindly provided by Dr. Steven Xu at the Northern Plains Crop Science Laboratory of the USDA-ARS, North Dakota, USA. The tobacco line NC89 was kindly provided by Dr. Xinwu Pei at the Biotechnology Research Institute of the Chinese Academy of Agricultural Sciences. Three diploid accessions of wild species of wheat, PI428182 (*Triticum urartu*, AA, 2n = 14), PI554296 (*Aegilops speltoides*, SS, 2n = 14), and TD125 (*Aegilops tauschii*, DD, 2n = 14), were kindly granted by Prof. Yueming Yan at Capital Normal University, in Beijing, China.

### Isolation of DNA and RNA and polymerase chain reaction (PCR)

Plant genomic DNA (gDNA) was isolated and purified with a standard CTAB method^43^. Gel electrophoresis was performed to check gDNA integrity and quantity. Total RNA was extracted from plant leaves using TRIzol reagent (Invitrogen, USA) according to the manufacturer’s instructions. Complementary DNA (cDNA) was synthesized using a Takara reverse transcription kit. Primers for *TaVIP2* cloning, chromosome location, and subsequent identifying positive transgenic tobacco plants were designed by Primer 5 software and synthesized (Sangon, China) (see Supplementary Table S1). The 20-μl PCR amplification reaction mixture contained 10 × PCR buffer, 1.5 mM MgSO_4_, 200 μM of each dNTP, 0.5 μM of each primer, 1 U Taq (Takara), and 100 ng of template cDNA or gDNA. PCR amplification was performed on a Bio-Rad DNA thermal cycler (model C1000). The PCR thermocyling program started from denaturation at 95 °C for 10 min, followed 32–35 cycles at 94 °C for 30 sec, 60–62 °C for 30 sec, and 68–72 °C for 1.5 min, and terminated by a final extension step at 72 °C for 5 min. PCR products were separated on 0.8% agarose gels and visualized with a Genecolour DNA Staining II TM (Gene-Bio Ltd). qRT-PCR primers were designed according to the conserved nucleotide sequences; these are detailed in Supplementary Table S1. A 20-μL reaction volume (SYBR PrimeScript RT-PCR Kit, Takara) containing 10 μl 2 × SYBR Premix Ex Taq, 2 μl first-strand cDNA, 0.3 μl primer mix (10 μM), 0.4 μl ROX Reference DyeII, and 7.3 μl ddH_2_O were used. qRT-PCR was performed with an ABI PRISM 7500 Real-Time PCR System (ABI, USA) with a thermocycling program of 95 °C for 30 sec, followed by 40 cycles of amplification (95 °C for 5 sec, 60 °C for 20 sec, 72 °C for 20 sec). qRT-PCR results were analyzed using the vendor PCR system software. Tobacco *NtActin* was used as a reference gene for normalization of expression values. All reactions were conducted with three biological replicates. Statistical analysis of the qRT-PCR data was performed with DPS software (IBM, USA).

### Southern blot analysis

Southern blot analysis was performed to determine the copy number and chromosome location of *TaVIP2* in wheat and to detect the presence of transgenic insertions in transgenic tobacco plants. 10 μg of genomic DNA was digested separately with four selected restriction enzymes (*Eco*RV, *Sac*I, *Dra*I, and *Kpn*I for the digestion of wheat genomic DNA, and *Hin*dIII for the digestion of transgenic tobacco DNA (Takara)). The digested products were fractionated with 0.8% agarose gel electrophoresis and transferred onto a nylon Hybond-N + membrane (Roche) with a membrane transfer machine (Model 785, Bio-Rad). The full length cDNA of *TaVIP2*, labeled with Digoxigenin, was used as a probe to hybridize with the digested genomic DNA. Hybridization and other steps were performed according to the manufacturer’s instructions for the DIG High Prime DNA Labeling and Detection Starter Kit II (Roche).

### Tobacco transformation

Sterile tobacco leaves (NC89) from tissue culture plants were cut into 1 cm × 1 cm pieces and pre-cultured on MS solid medium (Sigma)) (3% sucrose and 0.8% agar) for 3 days in the dark. *Agrobacterium* strain C58C1, which harbors the recombination vector with *TaVIP2*, was grown at 28 °C overnight in YEP medium (1% tryptone), 1% yeast extract, and 0.5% NaCl) containing 100 mg L^−1^ kanamycin, 100 mg L^−1^ gentamycin, and 50 mg L^−1^ rifampicin (all chemicals obtain from Sigma). When the OD_600_ value reached 0.6, the bacterial solutions were centrifuged at 3,500 rpm in a benchtop centrifuge for 10 min. The pellet was re-suspended in liquid MS medium containing 2 × MS mineral salts. The pre-cultured tobacco tissues were soaked in the *Agrobacterium* suspensions for 20 min, dried on sterile filter paper to remove excessive bacteria, then co-cultured for 3 days on solid MS medium in the dark at 25 °C. Putative transgenic shoots were regenerated on MS medium containing 100 mg L^−1^ kanamycin and 250 mg L^−1^ carbenicillin after 3 weeks culture in the light at 25 °C. The generated tobacco multiple shoots were transferred onto rooting medium (1/2 MS medium containing 100 mg L^−1^ kanamycin and 250 mg L^−1^ carbenicillin). Transgenic tobacco plants were verified by PCR, and the transgene expression level in positive plants was measured by qRT-PCR with primers specific for *TaVIP2*.

PCR-confirmed positive tobacco transgenic T_0_ plants with a single or two copies of *TaVIP2* integration were propagated via tissue culture using fresh leaf discs on MS medium containing 100 mg L^−1^ kanamycin prior to use in subsequent retransformation and powdery mildew resistance assays in a greenhouse. The procedure for retransformation was the same as the aforementioned transformation method for tobacco, with the exception that 5 mg L^−1^ of bialaphos (Wako) was added in the selection medium to replace 100 mg L^−1^ kanamycin after the co-cultivation step. Retransformation efficiencies of different transgenic tobacco plants with *TaVIP2* were calculated based on the results of PCR genotyping experiments.

### Powdery mildew resistance assays

Transgenic tobacco and control plantlets were transplanted into pots and grown in a greenhouse. At the four- to five-leaf stage, all plants were artificially inoculated with the causal pathogen of tobacco powdery mildew. About one month later, the powdery mildew resistance of each plant was evaluated based on the national criteria of China (GB/T 23222-2008, including six grades: 0, 1, 3, 5, 7, and 9, which correspond, respectively, to disease incidence levels of 0, ≤ 5%, 6–10%, 11–20%, 21–40%, and ≧ 41%).

## Additional Information

**How to cite this article**: Zhao, P. *et al*. Cloning and characterization of *TaVIP2* gene from *Triticum aestivum* and functional analysis in *Nicotiana tabacum*. *Sci. Rep*. **6**, 37602; doi: 10.1038/srep37602 (2016).

**Publisher’s note:** Springer Nature remains neutral with regard to jurisdictional claims in published maps and institutional affiliations.

## Supplementary Material

Supplementary Information

## Figures and Tables

**Table 1:** Retransformation efficiency of the transgenic tobacco plants expressing *TaVIP2*, based on PCR analysis.

Tobacco type	Leaf discs transformed	Positive plants obtained	Transformation efficiency (%)
TV2-11^#^	33	31	93.9^**^
TV2-49^#^	21	17	81.0^**^
Control	87	33	37.9

^#^Different transgenic tobacco lines expressing *TaVIP2*.

*Indicates significant differences at the P < 0.01 level.
