# Supplementary material for: Cloning and characterization of TaVIP2 gene from Triticum aestivum and functional analysis in Nicotiana tabacum
Source: Sci Rep. 2016 Nov 18;6:37602. doi: 10.1038/srep37602 (PMC5114603; doi:10.1038/srep37602)
Supplement: Supplementary Information [file srep37602-s1.pdf]

Cloning and characterization of *TaVIP2* gene from *Triticum aestivum* and functional analysis in *Nicotiana tabacum*

Pei Zhao [¶], Ke Wang [¶], Zhishan Lin [¶], Wei Zhang, Lipu Du, Yunlong Zhang & Xingguo Ye [*]

Institute of Crop Science, Chinese Academy of Agricultural Sciences, Beijing 100081, P.R. China

[¶] Contributed equally to this work.

[*] Corresponding author: Xingguo Ye

Address: 12 Zhong Guan Cun South St, Haidian District, Beijing 100081, China; E-mail: yexingguo@caas.cn; Phone: 86-10-8210-5173; Fax: 86-10-8210-8789

Supplementary material

Supplementary Fig. S1 Isolation of putative *TaVIP2* gene in common wheat. The left lane is the DNA marker, and the right lane is 1900 bp amplified fragment of *TaVIP2* gene.

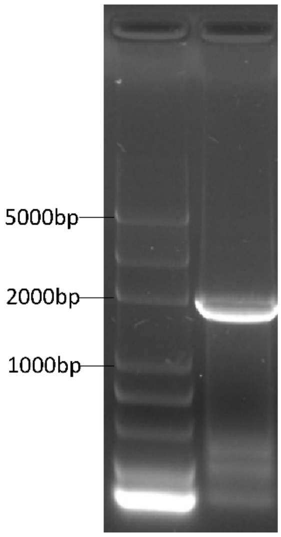

Supplementary Fig. S2 Chromosomal location of the three homoeoalleic *TaVIP2* genes in wheat via AS-PCR. a: The two sets of durum wheat substitution lines of 1D(1A) to 7D(7A) and 1D(1B) to 7D(7B) were amplified by the specific primers to the allele on A genome, and substitution lines of 2D(2A) to 7D(7A) and 1D(1B) to 7D(7B) as well as Langdon showed the 802bp specific band except substitution line 1D(1A). b: Amplified by the specific primers to the allele on D genome, only substitution lines 1D (1A) and 1D(1B) showed the 447bp specific band and the other lines as well as Langdon did not display the band. c: Amplified by the specific primers to the allele on B genome, 1D(1A) to 7D(7A) and 2D(2B) to 7D(7B) as well as Langdon appeared the 499bp specific band except substitution line 1D(1B). M represents 2000bp plus marker. Samples 1-15 stands for 1D(1B), 2D(2B), 3D(3B), 4D(4B), 5D(5B), 6D(6B), 7D(7B), Langdon, 1D(1A), 2D(2A), 3D(3A), 4D(4A), 5D(5A), 6D(6A), and 7D(7A), respectively.

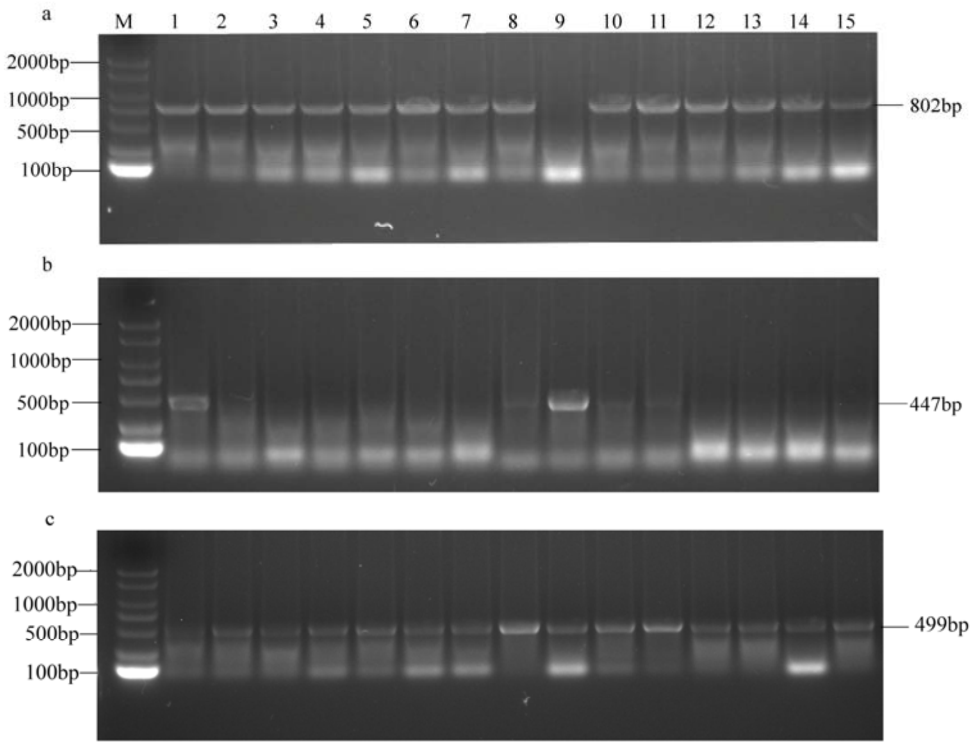

Supplementary Fig. S3 cDNA sequence comparison of the three copies of *TaVIP2* from the A, S(B), and D genomes.

The single nucleotide polymorphisms (SNPs) among the three *TaVIP2* genes are marked with red frames.

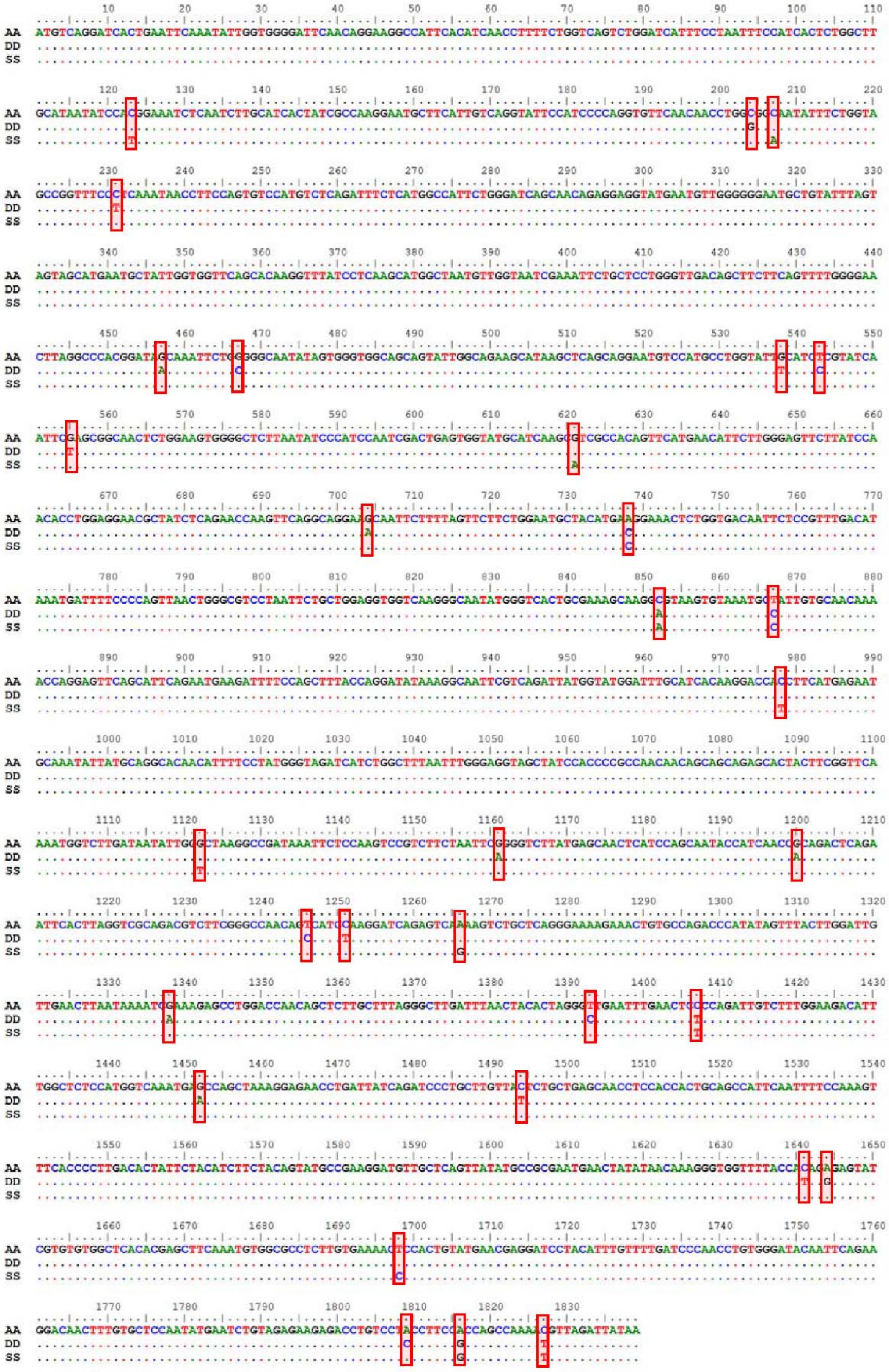

Supplementary Fig. S4 Phylogenetic analysis of 29 plant *VIP2* genes.

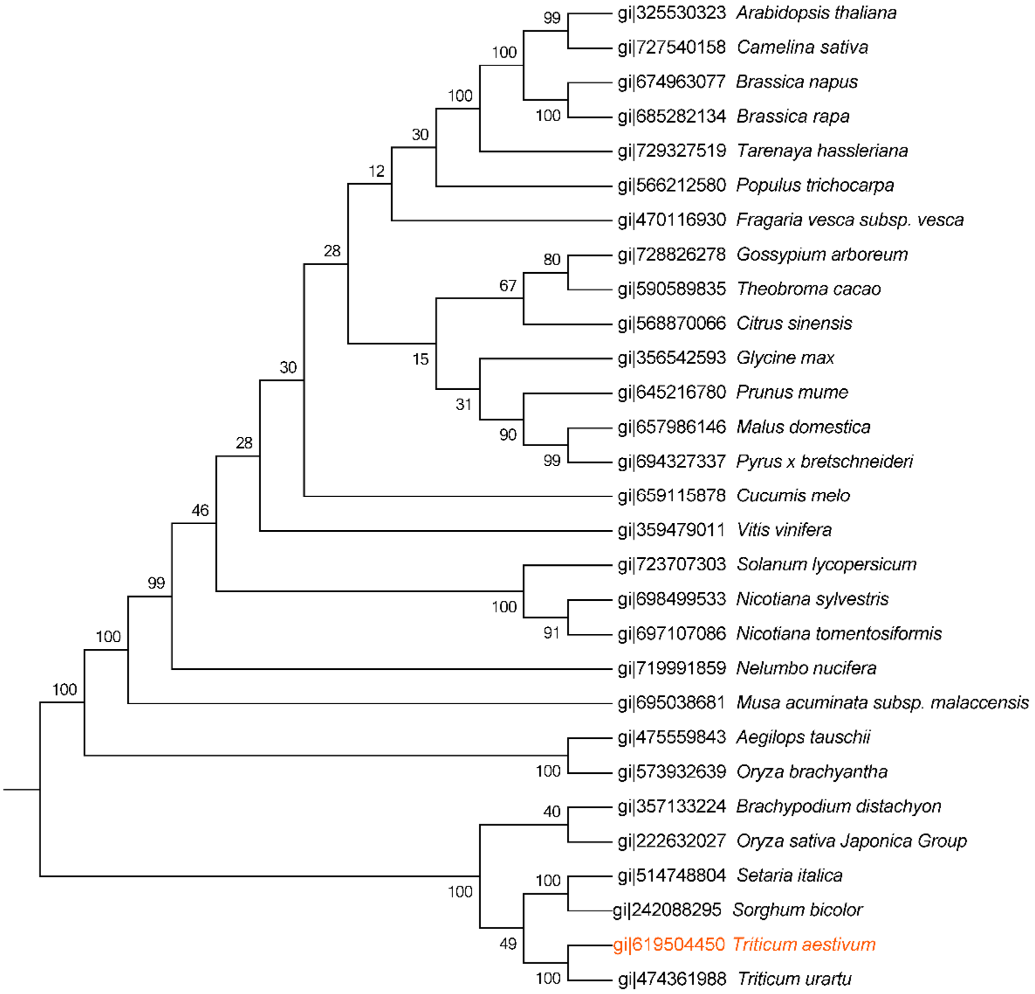

Supplementary Fig. S5 The modified pBI121-*TaVIP2* expression vector

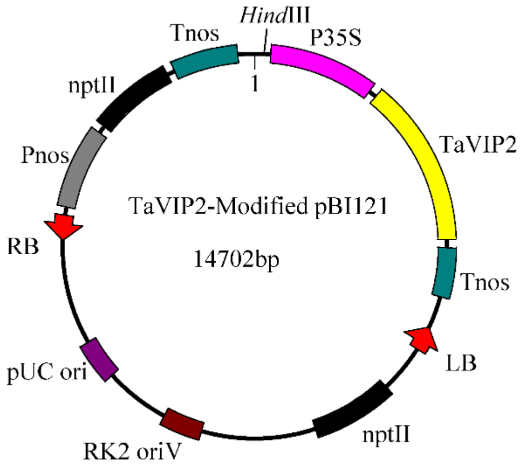

Supplementary Fig. S6 Detection of the *TaVIP2* gene in transgenic tobacco plants by PCR. M stands for DNA marker, lanes 1-4, 6, and 8-10 for positive plants, lanes 5, 7, and 11 for negative plants, and lane 12 for a wild type plant. The positive plants showed had a 666 bp amplified product of *TaVIP2*.

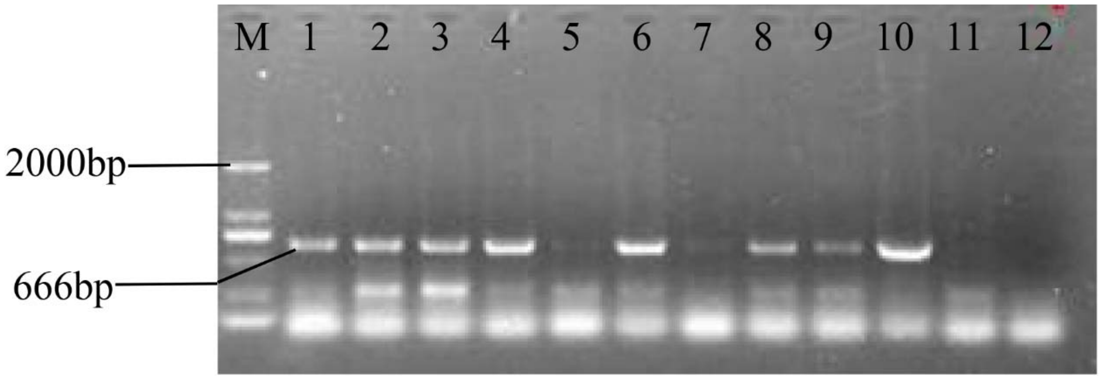

Supplementary Fig. S7 The pWMB178 expression vector used for the re-transformation of transgenic tobacco.

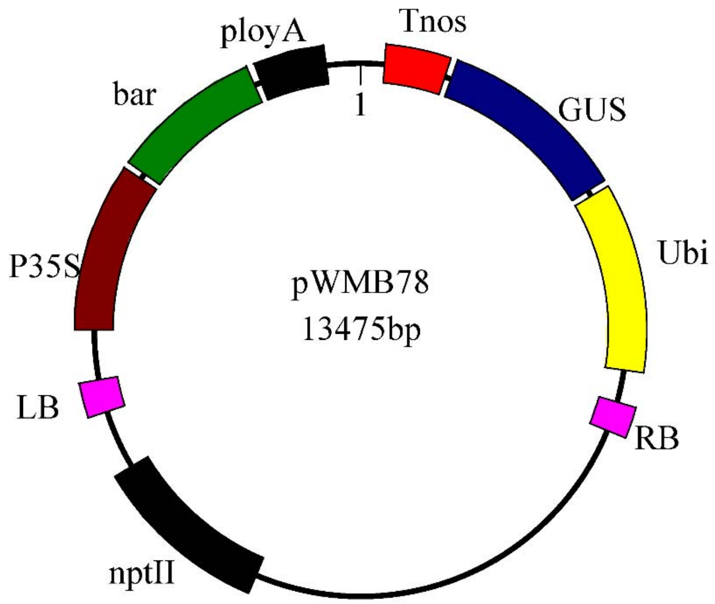

Supplementary Fig. S8 Production of shoots from transgenic tobacco plants overexpressing *TaVIP2* after re-transformation. a: Transgenic shoot regenerated from *TaVIP2* overexpression transgenic tobacco; b: Transgenic shoot regenerated from negative transgenic tobacco.

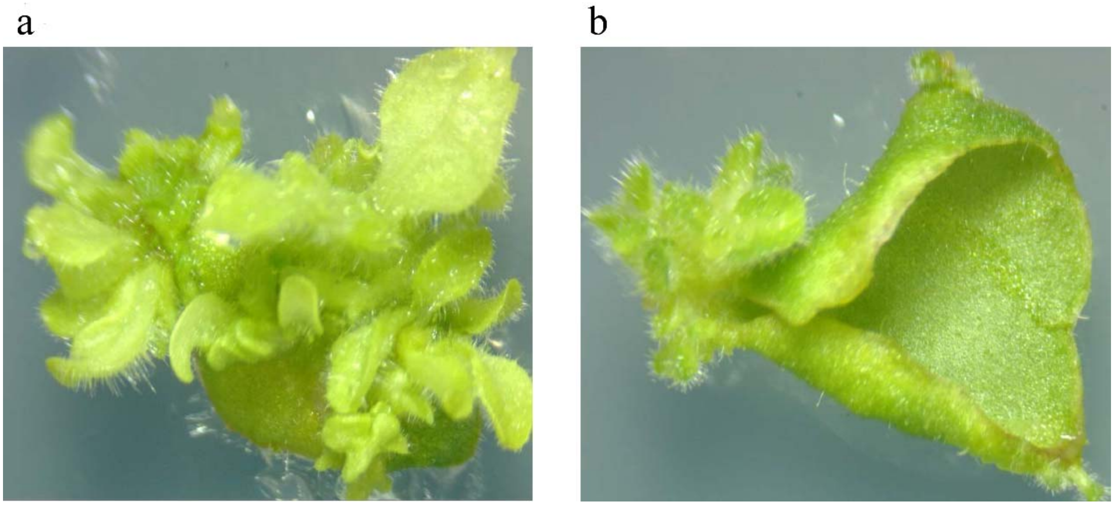

Supplementary Fig. S9 PCR analysis of transgenic tobacco plants confirming the presence of the *bar* gene after re-transformation. M stands for DNA marker, lane 1 for plasmid with *bar*, lane 3, 4, 5 and 6 for positive transgenic tobacco plants, lane 2 for a non-transformed tobacco plant, lane 7 for a negative transgenic tobacco plant as negative control, and lane 8 for ddH_2O.

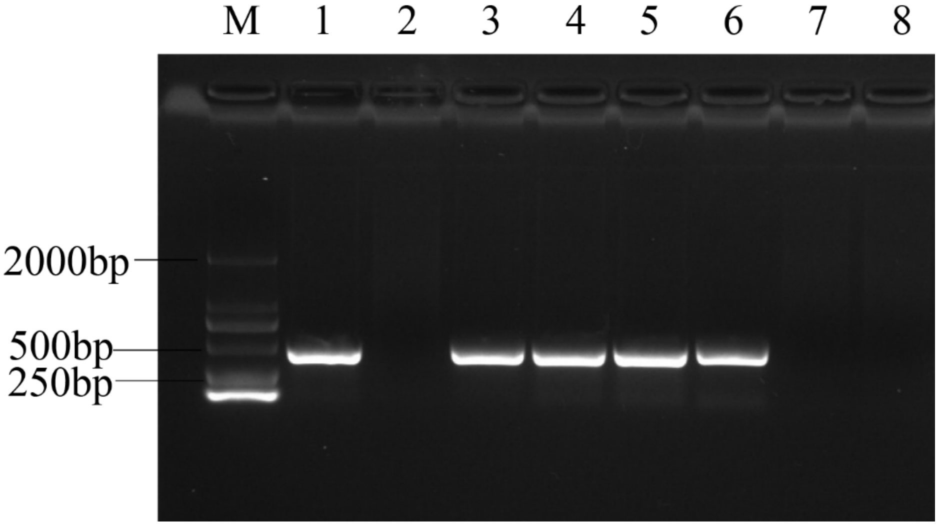

Supplementary Fig. S10 Expression level of some pathogen resistance-related genes in the transgenic tobacco plants expressing *TaVIP2*. Samples 1, 2, 3, 4, 5, and 6 represent the *TaVIP2* transgenic tobacco lines TV2-11, TV2-12, TV2-13, TV2-14, TV2-21, and TV2-49, respectively, and CK stands for the wild-type tobacco plant.

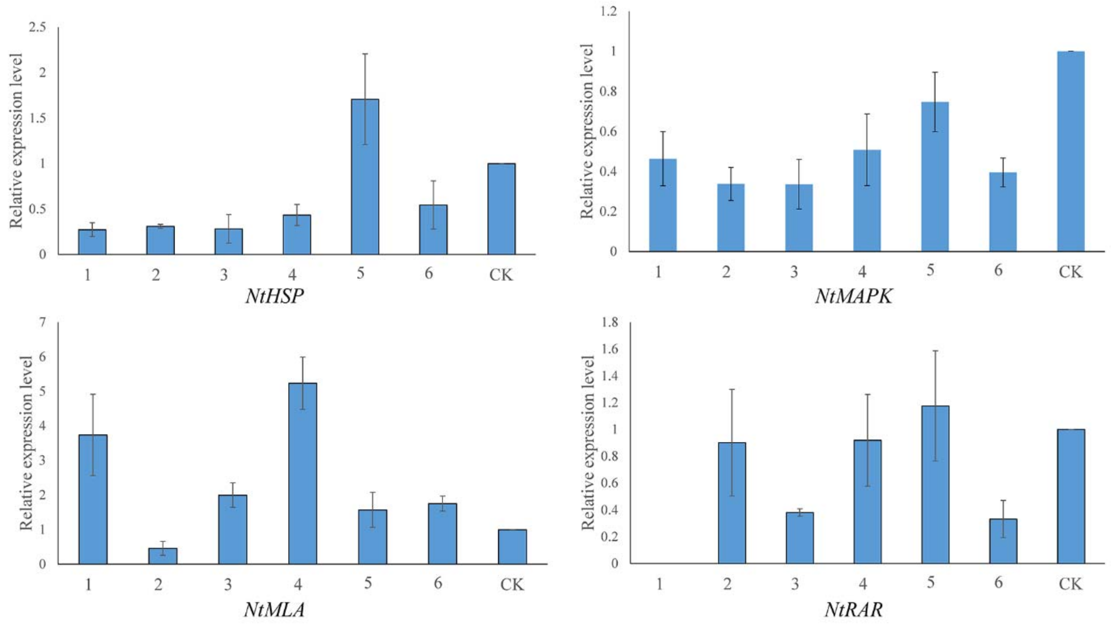

Supplementary Table S1 Sequences and purpose of all the primers.

Name	Forward primer(5'-3')	Reverse primer(5'-3')	Purpose
WV	AGAGGTACCATGTCAGGATCACTGAAT	TTATAATCTAACGTTTTGGCTGG	Cloning of *TaVIP2*
WNV	CAGCAGAGCACTACTTCGGT	ACAGGTCTCTTCTCTACAGATTCAT	Transgenic detection of *TaVIP2*
TaVIP2-A1	TAGCCTCTTGAACCCTGACATC	AAGATCTCGCAACCAACTCATG	Chromosome location of *TaVIP2* on A genome
TaVIP2-B1	TGGAATTACAGCTGAGAAGTTCTTG	TCCTCGTTCATACAGTGGAGTTT	Chromosome location of *TaVIP2* on B genome
TaVIP2-D1	CATTTCAGTCATATGACAGCGC	CCGTCATGTAGCATTCCAGAG	Chromosome location of *TaVIP2* on D genome
WBF	ACCATCGTCAACCACTACATCG	ACCATCGTCAACCACTACATCG	Transgenic detection of *bar*
TaVIP2	AGGAACGCTATCTCAGAACCAA	AGCATTTACACTTACGCCTTG	qRT-PCR
NtPR-1	TCGGTTCGTGTTGGATGT	TGGACGTAGGTCGTTTCA	qRT-PCR
NtHSP	CTCGGAACCACTTACTCTT	TCCTACCAATCAGCCTTT	qRT-PCR
NtMLA	GATTTGGGTGAACTATGC	ACTGTCCGCAATACTTAC	qRT-PCR
NtMAPK	GTGGCTTGAAGTATCTCC	TAAACTGCCCATTGTCTC	qRT-PCR
NtRAR	ATGATGGGATGAAGAAGTG	TAGAAGTTGGTGCTGGAA	qRT-PCR

Supplementary Table S2 The lengths of exons and introns of the T*aVIP2* genes from three wheat wild relative species.

No.	AA		DD		BB(SS)	
	Length of exon (bp)	Length of intron (bp)	Length of exon (bp)	Length of intron (bp)	Length of exon (bp)	Length of intron (bp)
1	18	324	18	326	18	325
2	88	89	88	202	88	89
3	155	88	155	86	155	88
4	358	645	358	688	358	688
5	213	88	213	87	213	88
6	102	458	102	577	102	459
7	86	82	86	82	86	78
8	75	155	75	160	75	157
9	426	76	426	75	426	77
10	53	75	53	75	53	75
11	42	718	42	547	42	626
12	145	85	145	86	145	86
13	78		78		78	
